# Neural underpinnings of threat bias in relation to loss-of-control eating behaviors among adolescent girls with high weight

**DOI:** 10.3389/fpsyt.2023.1276300

**Published:** 2023-10-27

**Authors:** Meghan E. Byrne, Marian Tanofsky-Kraff, Lucrezia Liuzzi, Tom Holroyd, Megan N. Parker, Bess F. Bloomer, Allison Nugent, Sheila M. Brady, Shanna B. Yang, Sara A. Turner, Daniel S. Pine, Jack A. Yanovski

**Affiliations:** ^1^Section on Development and Affective Neuroscience, Emotion and Development Branch, National Institute of Mental Health (NIMH), Bethesda, MD, United States; ^2^Division of Intramural Research, National Institutes of Health (NIH), Section on Growth and Obesity, Eunice Kennedy Shriver National Institute of Child Health and Human Development (NICHD), Bethesda, MD, United States; ^3^Department of Medical and Clinical Psychology, USUHS, Bethesda, MD, United States; ^4^Military Cardiovascular Outcomes Research (MiCOR) Program, Department of Medicine, Uniformed Services University of the Health Sciences (USUHS), Bethesda, MD, United States; ^5^MEG Core Facility, National Institute of Mental Health (NIMH), Bethesda, MD, United States; ^6^Nutrition Department, Clinical Center, NIH, Bethesda, MD, United States

**Keywords:** loss-of-control eating, obesity, adolescents, attention bias, binge-eating, magnetoencephalography

## Abstract

**Introduction:**

Loss-of-control (LOC) eating, a key feature of binge-eating disorder, may relate attentional bias (AB) to highly salient interpersonal stimuli. The current pilot study used magnetoencephalography (MEG) to explore neural features of AB to socially threatening cues in adolescent girls with and without LOC-eating.

**Methods:**

Girls (12–17 years old) with overweight or obesity (BMI >85th percentile) completed an AB measure on an affective dot-probe AB task during MEG and evoked neural responses to angry or happy (vs. neutral) face cues were captured. A laboratory test meal paradigm measured energy intake and macronutrient consumption patterns.

**Results:**

Girls (*N* = 34; *M*_age_ = 15.5 ± 1.5 years; BMI-*z* = 1.7 ± 0.4) showed a blunted evoked response to the presentation of angry face compared with neutral face cues in the left dorsolateral prefrontal cortex, a neural region implicated in executive control and regulation processes, during attention deployment (*p* < 0.01). Compared with those without LOC-eating (*N* = 21), girls with LOC-eating (*N* = 13) demonstrated a stronger evoked response to angry faces in the visual cortex during attention deployment (*p* < 0.001). Visual and cognitive control ROIs had trends suggesting interaction with test meal intake patterns among girls with LOC-eating (*p*s = 0.01).

**Discussion:**

These findings suggest that girls with overweight or obesity may fail to adaptively engage neural regions implicated in higher-order executive processes. This difficulty may relate to disinhibited eating patterns that could lead to excess weight gain.

## 1. Introduction

Approximately one-third of US youth have overweight and 17% have obesity (1–4); these conditions often persist during adulthood (5–8). The DSM-5 definition of binge-eating disorder (BED) (9) involves the presence of objectively large binge episodes with loss-of-control (LOC) overeating (defined as the subjective experience of being unable to stop eating) that occurs at least once a week for 3 months, where the episodes also have other characteristics, such as rapid eating, eating when not hungry or until feeling uncomfortably full, and/or feeling disgusted with oneself, depressed, or guilty after overeating. Full syndrome BED is less common during adolescence than adulthood (10). However, people reporting even low-frequency episodes of LOC-eating (without objectively large binge episodes) share many of the characteristics found among those who have BED (11). For example, LOC-eating uniquely places youth at risk for overweight and obesity (12, 13). Regardless of age or disordered eating status, individuals with obesity also report greater interpersonal stress, characterized by frequent and pervasive weight-related stigma (14–16), than individuals having lower weight (17). Theories of interpersonal sensitivity (18, 19) posit that pervasive experiences of social criticism or exclusion (e.g., weight stigma) increase awareness and vigilance toward negative feedback cues. Indeed, individuals with obesity display increased sensitivity to negative social feedback as indicated by an attention bias to social threat (20, 21). Furthermore, adolescents (22), girls in particular (23, 24), exhibit heightened vulnerability to social stressors. Given the frontal cortex is not yet fully developed during adolescence, this neural region may be particularly vulnerable to psychosocial stress during this stage of development (25). According to interpersonal theory, LOC-eating results from maladaptive coping with social stress (26–30), for example, social exclusion or criticism due to weight stigma. Maladaptive coping, in turn, might reflect perturbed neural responses to social cues (31, 32). Indeed, neuroimaging data support interpersonal theory in revealing that girls with overweight or obesity and LOC-eating express aberrant responses to social distress (i.e., simulated peer rejection) in the ventral prefrontal cortex, striatum, and fusiform face area (31), supplying several regions of interest (ROIs) for analyses. Taken together, interpersonal theory and extant neuroimaging data suggest that girls with LOC-eating may manifest abnormal neural responses to social threats. The current study utilizes magnetoencephalography (MEG), a novel brain imaging tool, to extend this previous study.

Neural correlates of LOC-eating may manifest within attention tasks that employ social threats. Attention bias, expressed as an excessive response to such threats, relates to many health conditions, such as disordered eating (33, 34). In conjunction with the interpersonal model of disordered eating, youth with LOC-eating may respond abnormally to attention paradigms, which engage two key temporal processes: automatic or “bottom-up” attention and voluntary or “top-down” attention. Automatic attention capture involves the engagement of ventral–frontal networks encompassing the insula, anterior cingulate, and medial prefrontal cortices (35–37), which typically occur within 250 milliseconds (ms) (32, 38). Voluntary attention capture involves the engagement of dorso-frontal regions encompassing the dorsolateral prefrontal cortex and dorsal anterior cingulate, which occur in a later or more sustained fashion (35–37, 39, 40). This offers several additional ROIs for imaging (i.e., insula, anterior cingulate, and medial and dorsolateral prefrontal cortices). Aberrant activation in these regions may fail to regulate attention deployment in ways that unleash attentional bias to social threats. Previous research studies have shown strong engagement of occipital regions in healthy individuals during the processing of negative-valence emotional stimuli (41–43), supplying another ROI for a visual attention-biased social threat task.

Imaging studies may extend the interpersonal model of LOC-eating. Circuitry central to food reward processing overlaps with ROIs from attention biased imaging research (44–48). Individuals with disordered eating manifest hyperactivity in regions supporting attention capture and social threat processing, such as the amygdala and anterior cingulate cortex (49–51), supplying additional ROIs for analyses. One relevant functional magnetic resonance imaging (fMRI) study examined 22 adolescent girls with overweight and obesity, finding that, following a social rejection task, girls with reported LOC-eating failed to engage prefrontal cortex regions implicated in emotion regulation and demonstrated hyperactivity in the fusiform face area, which subsequently related to palatable food intake at a laboratory test meal (31). fMRI, however, is limited in its capacity to elucidate temporal sequences of neural activity. Neural processes involved in attentional bias occur over a short period of time; these processes are optimally measured by temporally sensitive methods (36). MEG, which has both excellent temporal and acceptable spatial resolution (52–54), has been used to isolate neural response periods of attention bias to social threat in pediatric samples (55). This provides a precise quantification of attentional bias to psychosocial stress as a potential mechanism for LOC-eating.

The current study examined the links between neural activation implicated in attention bias to social threat and subsequent laboratory test meal energy intake. The sample consisted of adolescent girls with overweight or obesity, with and without reported LOC-eating. We hypothesized that, compared with neutral control face cues, youth would exhibit a greater bottom-up evoked response to social threat cues in aforementioned ROIs related to attention capture and threat detection [i.e., occipital, amygdala, fusiform, insula, striatum, anterior cingulate cortex (ACC), ventrolateral prefrontal cortex (PFC), medial PFC, and dorsolateral PFC]. Similarly, this sample of youth with overweight or obesity was expected to exhibit a blunted top-down evoked response to angry salient face cues in ROIs related to attention deployment. Moreover, youth with LOC-eating were expected to exhibit a greater bottom-up evoked response and a blunted top-down evoked response to social threat cues compared with youth without LOC-eating. Finally, it was hypothesized that the associations between bottom-up evoked response to angry face cues and maladaptive test meal intake patterns [i.e., greater total calorie intake and percentage of consumption from carbohydrates and fats and lower percentage of intake from protein, in line with previous research studies (56)] would be most strongly positively associated with girls with LOC-eating. The relationships between top-down evoked response and maladaptive test meal intake patterns were expected to be most strongly negatively related to girls with LOC-eating.

## 2. Materials and methods

### 2.1. Participants and recruitment

The inclusion criteria of the participants were as follows: female sex, 12–17 years old at the start of the study, overweight or obesity (BMI at or above the 85th percentile for age and sex according to the Centers for Disease Control US standards) (57), English-speaking, and right-handedness. Girls were excluded if they met any of the following criteria: an obesity-related health comorbidity requiring medical treatment, such as hypertension or fasting hyperglycemia consistent with diabetes; presence of other major medical illnesses including renal, hepatic, gastrointestinal, endocrinologic, hematological problems, or pulmonary disorders; regular use of any medication known to affect body weight or eating behavior (e.g., stimulants prescribed for attention deficit hyperactivity disorder); current pregnancy or a history of pregnancy; a significant reduction in weight during the past 3 months, for any reason, exceeding 5% of body weight; presence of a significant, full-threshold psychiatric disorder based on the DSM-5 (9) criteria that may impede competence or compliance or possibly hinder completion of the study; a history of significant or recent brain injury that may considerably influence performance; current involvement in a weight loss program, participating in psychotherapy aimed at weight loss or treatment of eating behavior; or a condition under which MEG participation would be contradicted (e.g., metal in the body, pregnancy, claustrophobia, and history of significant neurological insult or injury). Girls who reported allergies to gluten, nuts, dairy, fruit, or any other item in the test meal array were excluded from the test meal portion of the study.

Participant recruitment methods involved mailing to families in the greater Washington, D.C. metropolitan area. This recruitment method has been used successfully for prior community-based studies of youth with and without LOC-eating (13, 56, 58). All participants received monetary compensation for each visit. Based on rates reported in previous studies involving fMRI and MEG in youth, 35% attrition was estimated a priori due to excessive movement during MRI/MEG (55, 59). Consistent with standards in the disordered eating literature (10, 56, 60), LOC-eating was defined by the presence of one or more episodes of LOC-eating during the previous month prior to assessment, as assessed using a clinical diagnostic interview for eating disorders (61) described below.

### 2.2. Study procedures

The National Institutes of Health (NIH) Institutional Review Board (IRB) approved all study procedures. All study visits took place at the NIH Hatfield Clinical Research Center. The current study was a component of the Pilot Mobile Attention Retraining in Overweight Female Adolescents protocol (ClinicalTrials.gov ID: NCT02977403). All data for the current study were collected prior to the participation of youths in the larger protocol's intervention. Adolescent girls with overweight or obesity participated in a screening visit and a laboratory visit. At the first visit, interested parents and daughters signed IRB-approved consent and assent forms, and then, participants underwent a physical examination with weight and height objectively measured in triplicate by trained staff and two semi-structured interviews to determine eligibility. Total mass (kg), lean mass (kg), fat mass (kg), and fat mass percentage (%) were assessed by dual-energy x-ray absorptiometry (DXA, GE Lunar iDXA, GE Healthcare, Madison WI; software GE encore 15), a validated measure of body composition in youth (62). During the laboratory visit, girls completed an anatomical MRI scan, a social threat attention bias paradigm while undergoing MEG, and lastly a laboratory test meal. Participants were instructed to begin fasting at 10:00 p.m., the night before the laboratory visit. At approximately 10:00 a.m. in the morning of the laboratory visit, participants consumed a standardized breakfast shake (17% protein, 16% fat, and 67% carbohydrate) containing 21% of daily energy needs as estimated by measured body weight, height, age, and average activity level within the previous week (63).

#### 2.2.1. Semi-structured interviews

##### 2.2.1.1. Eating disorder examination

The Eating Disorder Examination (EDE) (61) is a semi-structured diagnostic interview of eating disorder psychopathology. The EDE contains 21 items that assess disordered attitudes and behaviors related to cognitive and dietary restraint, eating, body shape, and weight, and 13 items designed and adapted to diagnose specific DSM-5 eating disorders. The presence or absence of LOC-eating was determined by the overeating subsection of the EDE. Girls who reported at least one episode of LOC-eating in the past 28 days on the EDE were categorized into the LOC-eating group. The EDE has demonstrated sound psychometric properties, including good construct validity with physiological and objective measures and good-to-excellent interrater reliability (Cohen's κ = 0.8–1.0) in adolescent samples (10, 64).

##### 2.2.1.2. Kiddie schedule for affective disorders and schizophrenia for school-age children

The Kiddie Schedule for Affective Disorders and Schizophrenia for School-Age Children (KSADS) (65) is a reliable and valid semi-structured diagnostic interview to assess DSM-5 psychiatric diagnoses. Specific diagnostic sections of the KSADS were used to assess psychiatric functioning and exclude participants with significant psychiatric comorbidities (e.g., bipolar, psychosis, and suicidality) that may have impeded competence or compliance or possibly hindered completion of the study. The KSADS was only administered to participants who positively endorsed depression, suicidal ideation, mania, psychosis, or substance use disorder on a screening measure. Girls were administered only a relevant portion of the KSADS in order to identify any current psychiatric issues that warranted the exclusion of the study or would have impeded adherence to the study.

#### 2.2.2. Anatomical MRI scan

An MRI scan was conducted on a Siemens MAGNETOM Verio 3T scanner equipped with a 16-channel head/neck coil for co-registration with MEG. Standard imaging parameters were used including the acquisition of T1-weighted structural images for co-registration. Individuals were screened for contraindications before the scan. In addition, all magnetic objects (e.g., watches, coins, and jewelry) were removed before entering the MRI scan room. As all participants were female, a pregnancy test was performed not more than 24 h before the structural MRI scan. Participants were fitted with hearing protection during the MRI scan. Anatomical MRI data preprocessing was conducted using AFNI software, which included removing the skull and normalizing it to standard Talairach coordinates.

#### 2.2.3. Magnetoencephalography equipment and procedures

This study was performed on the 275-channel CTF MEG^TM^ brain imaging system at the National Institute of Mental Health MEG CORE Facility. Participants sat in the magnetically shielded recording room with their heads positioned in the MEG helmet. The position of the head was determined before and after each MEG session by localizing the position of three indicator coils that are attached to the preauricular and the nasion fiducial points. The positions were used to co-register the MEG coordinate system with the individual MRI. Padding was used to minimize movement of the head during the scan. Participants with excessive head motion and poor MRI co-registration, according to visual inspection, were excluded from the MEG analysis. MEG data were sampled at 600 Hz, and synthetic 3^rd^-order gradiometer correction was applied to reduce environmental noise. The entire MEG session lasted for approximately 60 min.

MEG source reconstruction was performed in MNE Python (66) with a multiple-sphere head model and an LCMV beamformer on a 5-mm grid. ROI voxels were defined with Freesurfer (aparc.a2009s+aseg.mgz) with reference to individual MRIs. Sensor data were first filtered between 1 and 30 Hz and then segmented into trials with reference to the presentation of face stimuli (*t* = 0) in a time window starting 500 ms pre-stimulus and ending 800 ms post-stimulus. The data covariance was calculated from the concatenated data of all trials to estimate beamformer weights. The trial-average responses to each face stimulus type (angry, happy, or neutral) were then reconstructed to voxels within the ROIs. To account for the sign uncertainty of the beamformer inverse solution, responses for all voxels within an ROI were inverted to be positively correlated with each other. Evoked responses for each ROI were obtained as primary outcomes by averaging all voxels within the ROI.

#### 2.2.4. Social threat task

The social threat attention bias task involved two picture pairs of angry, happy, or neutral faces, presented on a computer screen, followed by a probe appearing behind one of the images, prompting a response. Normed face images were selected from the NimStim Face Stimulus Set (67). The task consisted of three face pair types, namely, neutral-neutral, happy-neutral, and angry-neutral. Each task block consisted of 15 face pairs of the same type, presented four times each. The order of pair-type blocks was randomized across all participants; however, the specific face pairs remained consistent. The location of each stimulus and probe was counterbalanced across participants. Importantly, the probe replaced the neutral images (i.e., neutral faces) and the salient images (i.e., happy or angry faces) with equal frequency. The design and contrasts of this social threat attention bias task have been used extensively in pediatric samples with anxiety symptoms and have been repeatedly shown to stimulate neural responsivity linked to regions associated with anxiety (32, 36, 55, 68, 69). Moreover, the task has been shown to stimulate similar neurocircuitry to that of other social anxiety laboratory paradigms (55, 69) and, in particular, circuits linked to previous research using fMRI among a sample of girls with overweight and obesity and LOC-eating (31). The use of angry faces, rather than disgust or fear social cues, is consistent with the previous imaging literature using the same attention bias dot-probe paradigm (55, 68, 70).

Participants completed a practice session before beginning the dot-probe task. The full task consisted of 180 trials divided into four segments with 45 trials each. Each pair of images appeared on the computer screen for 500 ms. After 500 ms, the image pair disappeared, and a probe (horizontal or vertical dots) appeared in one of the previously occupied photo locations for 200 ms. Participants were instructed to respond as quickly as possible with a right- or left-sided button to indicate the orientation of the dots. A central fixation cross appeared for 500 ms before each face pair presentation, and the blank inter-trial interval lasted for 1,300 ms. Trials consisted of a mixture of incongruent trials, in which the probe replaced the neutral face image, and congruent trials, in which the probe replaced the angry or happy face image. Sixty pairs of each combination (neutral-neutral, angry-neutral, and happy-neutral) were presented in randomized order. The spatial location of images and probes was counterbalanced. Trials were excluded from analyses if the participant did not respond within the time window or responded incorrectly.

#### 2.2.5. Laboratory test meal

**Figure 1:**
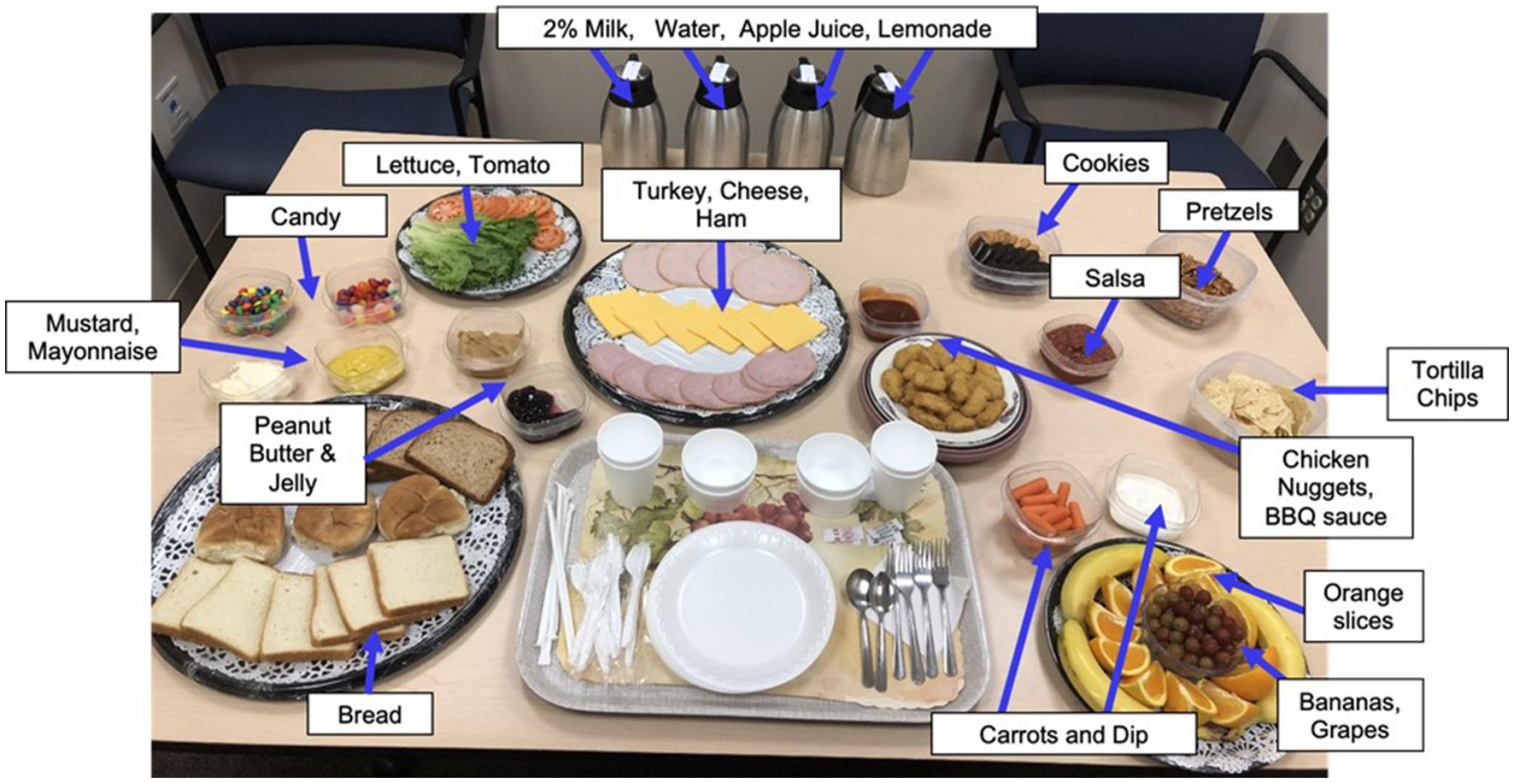
Laboratory buffet lunch test meal. Participants ate from a >10,000 kcal laboratory buffet lunch test meal consisting of a variety of macronutrients. Before the meal, all participants received tape-recorded binge instructions “Let yourself go and eat as much as you want.”

Immediately following the MEG scan, girls were introduced to the standardized multi-array test meal (~11,000 kcal, 12% protein, 33% fat, and 55% carbohydrate), which was designed to objectively capture LOC-eating behaviors involving excess calorie intake and consists of standard lunch-type foods, as well as those typically rated as highly palatable among youth (Figure 1) (56, 71). The test meal began with tape-recorded binge-eating instructions to “Let yourself go and eat as much as you want.” Participants notified the study team when they were finished eating. Consumption was calculated by weighing each food item before and after the meal. The primary outcome variables of interest were total energy (kcal) and percentage macronutrient (i.e., percentage of energy consumed from protein, fats, and carbohydrates) intakes. Energy content and macronutrient composition for each food item were determined according to the USDA National Nutrient Database for Standard Reference. This LOC-eating test meal paradigm is well-validated and has been successfully used in pediatric studies (56).

### 2.3. Statistical analyses

Analyses were performed using the Python 3 MNE package (66) and IBM SPSS Statistics (version 29). Descriptive statistics were calculated for participant demographic characteristics and laboratory test meal variables. Chi-square and independent samples *t-*tests were conducted to compare the groups of girls with and without LOC-eating on age, race, ethnicity, BMI-*z*, fat mass percentage, lean mass, and test meal consumption.

Behavioral attentional bias scores (i.e., “angry bias,” “happy bias”) were calculated by subtracting the mean reaction times in responding to probes replacing a salient stimulus (i.e., angry or happy face cues) from mean reaction times to probes replacing a neutral stimulus (i.e., neutral face cues), consistent with standards in prior literature (68, 72, 73). Reaction times are expected to be faster when the probe replaces a stimulus; the participant is currently orienting toward the time of the probe presentation onset. A bias score of 0 indicates no attentional bias to either cue, a negative score indicates a bias away from the more salient cue (i.e., angry or happy face), and a positive score indicates a bias toward the more salient cue. Paired-sample *t*-tests were conducted to examine whether behavioral attention bias scores differed by emotional salience (i.e., angry bias score and happy bias score) in the total sample. To examine whether angry and happy bias scores differ between youth with and without LOC-eating, a one-way analysis of variance (ANOVA) was conducted.

MEG analyses used a broad frequency band (1–30 Hz), in line with procedures from previous MEG studies (55, 74, 75). Nine neural ROIs (i.e., occipital, amygdala, fusiform, insula, striatum, ACC, ventrolateral PFC, medial PFC, and dorsolateral PFC) implicated in attention capture and orientation, threat detection, and executive control were selected *a priori* for analyses based on the previous literature (31, 37, 44, 46, 48–51, 76, 77). A complete list of ROIs and corresponding Freesurfer atlas labels is presented in Supplementary Table S1. Based on the previous literature (35, 36, 39) and visual inspection, the “bottom-up” initial attention capture period was defined as the time window between 0 and 250 milliseconds (ms) after each face stimulus onset, and the “top-down” maintained attention deployment period was defined as the time window between 250 and 600 ms after face stimulus onset. For the occipital region, the bottom-up time window is determined by visual inspection of peak evoked response at 140–250 ms rather than 0–250 ms, which is consistent with other research studies using visual regions of interest (76, 78). A significance threshold of *p* < 0.01 was applied to account for multiple comparisons. Only *a priori* ROIs that survived a *p* < 0.01 threshold were interpreted.

Paired-sample *t*-tests were conducted to examine whether the evoked response to face cues differed by emotional salience in the total sample (i.e., girls with and without LOC-eating). Comparisons of average evoked response during bottom-up (i.e., 0–250 ms) or top-down (i.e., 250–600 ms) time windows were examined between angry vs. neutral and happy vs. neutral face cues in each ROI. Next, ANOVAs were conducted to examine whether neural activity in ROIs differs between youth with and without LOC-eating. LOC-eating (presence/absence) was the independent variable (IV); bottom-up (i.e., 0–250 ms) or top-down (i.e., 250–600 ms) evoked response to angry, happy, or neutral face cue presentations in each ROI was examined as the dependent variable (DV).

To examine the association between angry or happy (vs. neutral) evoked response in ROIs and energy intake at the laboratory test meal, and whether LOC-eating moderated these associations, generalized linear mixed models were conducted. Arcsine square-root transformations were conducted for laboratory test meal percentage intake from carbohydrates, fats, and protein. Fixed model covariates included age, race, anthropometric variables (height, fat mass percentage, and lean mass), and pubertal status in order to account for body size, metabolic activity, and hormonal influences on outcomes (79, 80). IV was bottom-up (i.e., 0–250 ms) or top-down (250–600 ms) evoked responses to angry, happy, or neutral face cues in each ROI (i.e., occipital, amygdala, fusiform, insula, striatum, ACC, ventrolateral PFC, medial PFC, and dorsolateral PFC), and DV was energy intake variable (i.e., total kcal or % energy consumed from protein, fat, or carbohydrates). LOC-eating presence or absence served as an interaction term with evoked responses in ROIs, and age, race, pubertal status, height, fat mass percentage, and lean mass were included as covariates.

## 3. Results

### 3.1. Missing data and participant characteristics

It was estimated that 53 youth would have provided at least 80% power to detect small to medium effect sizes. Assuming a 35% attrition rate in unusable MEG data based on prior MEG studies in youth (55, 59), *N* = 80 youth were targeted for recruitment. However, due to the limitations of social distancing and stay-at-home orders during the COVID-19 pandemic, recruitment was truncated at *N* = 55 adolescent girls. All participants were female and had overweight or obesity (BMI ≥ 85^th^ percentile). In total, 21 (38%) of the 55 girls were missing MEG data due to several reasons, such as excessive (>80) task errors (*n* = 10), technical issues (*n* = 4), failure to administer the social threat task due to visit timing issues (*n* = 3), or poor anatomical co-registration (*n* = 4). Thus, there were *N* = 34 participants (average age of 15.5 ± 1.5 years) with complete and usable MEG data included in the final sample. Chi-square and *t-*tests revealed that there were no statistically significant differences between the groups of included and excluded girls on age, race, ethnicity, or percentage of energy consumed from proteins, carbohydrates, or fats at the laboratory test meal (*p*s > 0.08). Girls excluded from analyses were more likely to have obesity [*X*^2^ (1, N = 55) = 6.21, *p* = 0.01], had higher BMI-*z* scores [*t*(33) = 2.33, *p* = 0.02], and consumed a significantly greater total number of calories at the laboratory test meal [*t*(33) = 2.97, *p* = 0.01] compared with girls included in analyses.

Of the included participants, 13 (38.2%) had LOC-eating; 16 (47.1%) had obesity, and 18 (52.9%) had overweight; 12 (35.3%) participants identified as Black or African American; four (11.8%) identified as Hispanic or Latino; and the average BMI z-score was 1.7 ± 0.4. The characteristics of participants (*N* = 34) are presented in Table 1. Chi-square and *t-*tests revealed that there were no significant differences between the groups of girls with and without LOC-eating on age, race, ethnicity, BMI-*z*, body composition, bias scores, or percentage of energy consumed from proteins, carbohydrates, or fats at the laboratory test meal (*p*s > 0.09). Girls reporting LOC-eating consumed significantly more energy at the laboratory test meal compared with girls without LOC-eating [*t*(33) = −2.99, *p* = 0.01].

**Table 1:** Participant characteristics.

	**Total sample**	**LOC**	**No LOC**
	**(*****N*** = **34)**	**(*****N*** = **13)**	**(*****N*** = **21)**
Age (years)	15.5 ± 1.5	15.8 ± 1.3	15.3 ± 1.6
Race (%)		
White	44.1	46.2	42.9
Black or African American	35.3	53.8	23.8
Multiple Races	17.6	0.0	33.3
Ethnicity (%)		
Hispanic or Latino	11.8	0.0	19.0
Non-Hispanic or Latino	85.3	100.0	76.2
Not reported	2.9	0.0	4.8
BMI-*z*	1.7 ± 0.4	1.7 ± 0.5	1.6 ± 0.4
Fat mass (%)	40.8 ± 6.0	41.1 ± 7.3	40.7 ± 5.3
Lean mass (kg)	43.3 ± 6.0	45.7 ± 7.5	41.8 ± 4.5
Overweight (%)	52.9	46.2	57.1
Obesity (%)	47.1	53.8	42.9
Angry bias score	1.43 ± 28.48	−3.16 ± 28.98	4.27 ± 28.50
Happy bias score	0.98 ± 31.31	2.40 ± 36.18	0.10 ± 28.81
Test meal, total Kcal	882.7 ± 291.7	1049.0 ± 211.3^*^	769.0 ± 288.3
Test meal, % carb	48.9 ± 8.2	51.2 ± 8.6	47.2 ± 7.8
Test meal, % fats	36.9 ± 6.5	34.9 ± 6.5	38.2 ± 6.4
Test meal, % protein	14.3 ± 2.8	13.8 ± 3.1	14.6 ± 2.7

LOC, Loss-of-control eating; BMI-*z*, body mass index z-score; ^*^p < 0.01.

### 3.2. Behavioral results

Analyses examining the angry bias score vs. happy bias score in the total sample revealed no significant difference in behavioral reaction times to either emotional condition compared with neutral [*t*(33) = 0.57, *p* = 0.95]. An ANOVA comparing angry bias score between girls with (*N* = 13) and without (*N* = 21) LOC-eating revealed no significant behavioral reaction time difference between the groups [*F*(1, 32) = 0.54*, p* = 0.47]. Similarly, an ANOVA comparing happy bias score between the LOC-eating groups revealed no significant difference in reaction time among girls with LOC-eating compared with girls without LOC-eating [*F*(1, 32) = 0.04*, p* = 0.84].

### 3.3. MEG results

**Figure 2:**
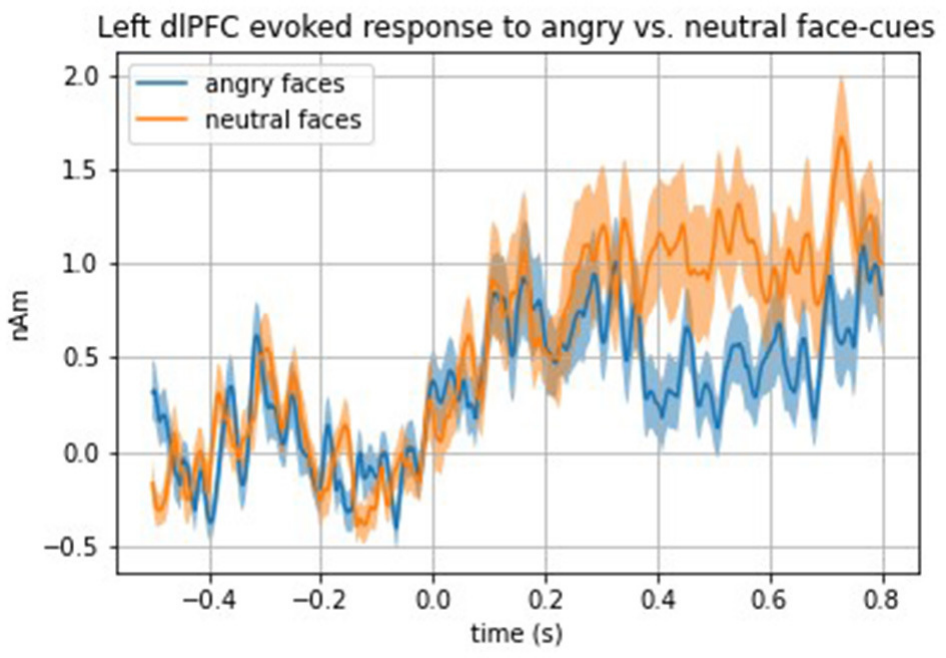
Time series of the total sample left dlPFC evoked response to angry vs. neutral face cues.

Analyses examining evoked responses to angry vs. neutral and happy vs. neutral faces in the total sample (i.e., girls with and without LOC-eating) revealed a significantly blunted (i.e., weaker) top-down (i.e., 250–600 ms time window) evoked response to angry faces compared with neutral faces in the left dorsolateral PFC (dlPFC) [*t*(33) = 2.80, *p* = 0.008] (see Figure 2 for time series of left dlPFC evoked response to angry face cues compared with neutral). No other top-down ROIs met the threshold for significance (*p*s > 0.01). All bottom-up findings were non-significant (*p*s > 0.02).

**Figure 3:**
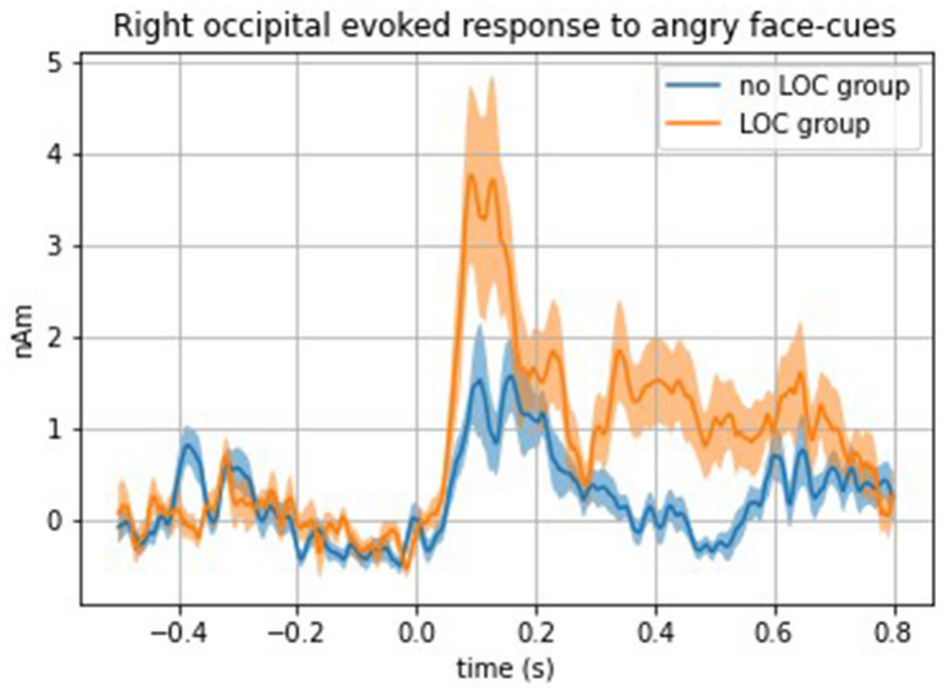
Time series of the right occipital evoked response to angry face cues among girls with and without LOC-eating.

Analyses comparing evoked response with face cues between girls with and without LOC-eating revealed a significantly stronger top-down evoked response to angry face cues in the right occipital cortex among girls with LOC-eating compared with girls without LOC-eating [*F*(1, 32) = 16.38, *p* = 0.0003] (see Figure 3 for time series of right occipital evoked response to presentation of angry face cues among girls with and without LOC-eating). No other top-down ROIs met the threshold for significance (*p*s > 0.01). All bottom-up findings were non-significant (*p*s > 0.02).

### 3.4. Test meal results

Models examining the main and interactional effects of angry, happy, or neutral evoked response with LOC-eating on test meal intake patterns are presented in Supplementary material. Models adjusted for all covariates (i.e., age, race, pubertal status, height, fat mass percentage, and lean mass). In response to happy face cues, a greater bottom-up evoked response to happy face cues in the right occipital cortex was marginally associated with a lower percentage of energy consumed from carbohydrates among girls with LOC-eating [*F*(1, 22) = 7.72, *p* = 0.01]. A greater top-down evoked response to happy face cues in the left dlPFC was marginally associated with a lower percentage of energy consumed from fats among girls with LOC-eating compared with girls without LOC-eating [*F*(1, 22) = 7.19, *p* = 0.01].

In response to angry face cues, a greater bottom-up evoked response in the left insula was marginally associated with a greater percentage of energy consumed from fats among girls with LOC-eating and a lower percentage of energy consumed from fats among girls without LOC-eating [*F*(1, 22) = 8.19, *p* = 0.01]. This finding was considered marginal due to the corrected *p*-value of 0.01. In addition, a greater top-down evoked response in the left ACC was marginally associated with a greater percentage of energy consumed from carbohydrates [*F*(1, 22) = 8.23, *p* = 0.01] and a lower percentage of energy consumed from fats among girls with LOC compared with girls without LOC-eating [*F* (1, 22) = 8.05, *p* = 0.01]. There were no significant interactions for evoked response to neutral cues, total calorie intake (kcal), or percentage of energy consumed from protein (*p*s > 0.01).

## 4. Discussion

The current study examined neural activation to social threat cues and subsequent energy intake at a laboratory test meal in girls with overweight or obesity. Three main findings emerged from the study. First, compared with those without LOC-eating, girls with LOC-eating demonstrated a stronger evoked response to angry faces in the visual cortex during attention deployment. Second, in this sample of girls with overweight and obesity examined as a whole, socially threatening cues, compared with neutral cues, elicited a blunted evoked response in the left dlPFC, a neural region implicated in executive control and regulation processes. Finally, inconclusive findings arose concerning relations between neural responses and energy intake patterns. While several notable trends were observed, they did not pass the threshold for statistical significance.

The findings revealed that a stronger visual neural response to social threat during attention deployment may differentiate youth with LOC-eating and overweight or obesity from those without LOC-eating. Such responses could involve both deleterious and protective effects. For example, prolonged visual attendance toward socially threatening stimuli may contribute to heightened social distress and subsequently trigger maladaptive coping via overeating (30), in line with interpersonal theory. Adolescent girls (22–24), as well as individuals with obesity across the lifespan (17), regularly confront a multitude of social stressors. As shown in a previous fMRI study among adolescent girls with overweight and obesity, social rejection was linked to failed engagement of prefrontal cortex regions implicated in emotion regulation, which subsequently related to palatable food intake at a laboratory test meal (31). Thus, the composition of the current sample, comprising adolescent girls with overweight, may include a group that is uniquely susceptible to interpersonal threat cues. Alternately, other facets of neural responding could mitigate risk. For example, a stronger initial visual response to positive cues may serve to protect against overeating of foods high in carbohydrates. Taken together, in line with interpersonal theory and the attentional bias literature outlined above, a visual attention bias to social threat may partially explain eating patterns among girls with obesity and LOC-eating. However, as mentioned below, the role of age and sex on these processes cannot be determined given the relatively homogenous sample.

Both the current and past studies suggest that girls with overweight or obesity manifest attention dysfunction when exposed to social threat cues. In this current sample of girls with overweight and obesity, socially threatening cues elicited a blunted response in the left dlPFC, a neural region implicated in executive control and regulation processes. Blunted engagement in the dlPFC could hinder cognitive efforts to efficiently deploy attention to mitigate overeating behaviors. This could represent a potentially modifiable mechanistic target (i.e., bolstering attentional awareness and control) for the prevention of excess weight gain. Effects of attention to a social threat on overeating could derive from influences on specific emotional processes. Indeed, previous research has shown that youth sometimes report “numbing” (60), or a blunted awareness of emotions, during LOC-eating episodes. Difficulty identifying emotional experiences (60, 81), also known as alexithymia, is also commonly reported during binge episodes among youth with LOC-eating. Attending to one's emotions facilitates one's ability to identify them; thus, aberrant emotion awareness may link weaker dlPFC engagement, observed among girls with LOC-eating compared with controls, to overeating. Accordingly, such relations among attention, emotion awareness, and the observed blunting in the dlPFC may further explain overeating behavior. The current findings may identify a potential neural basis for overeating among girls with LOC-eating and overweight or obesity.

Previous research demonstrates the importance of integrating findings from behavior and neural responses in brain imaging research. While between-group differences in neural responding arose in the current study, no such differences arose in behavioral reaction times. The recent literature has questioned the reliability of the behavioral attention bias score as a primary outcome of the dot-probe task (82, 83). Behavioral reaction time can be influenced by a variety of factors, such as response selection latency, that do not pertain to the underlying attentional bias neurocognitive construct (82). Moreover, behavioral results may have been subjected to a ceiling effect given the dot-probe task is not highly cognitively demanding. Such factors may explain the presence of stronger findings in the imaging than behavioral data. Behavioral manifestations of attentional bias likely arise from a multiplicity of causes. To best clarify the intersection of attentional bias and eating behavior, the underlying neural dysfunctions of attention bias must be disentangled using imaging approaches. These considerations will be important for future studies exploring the role of social threat in relation to overeating.

In the current study, stronger dlPFC response to salient positive (i.e., happy) social cues related to *lower* percentage consumption of fats among girls with LOC-eating. While replication of findings in larger samples is needed, the results could inform future research paradigms to retrain attention toward positive social cues. Bias toward positive social cues may conceivably be protective against overeating. Indeed, research supports the role of positive social engagement in the improvement of weight outcomes among adults (84), children (85), and adolescents (86, 87). A recent study demonstrated that thoughts about binge eating reduced following an interpersonal scenario characterized by social inclusion in a Cyberball task among individuals with BED (88), which aligns with current findings linking positive social cues and lower palatable food intake. Engagement of the dlPFC, which supports cognitive control processes, powerfully modulates engagement of the ventral stream, a series of brain regions beginning in the occipital cortex that represents the identity of objects. This includes emotionally salient objects, such as faces conveying threat detection (89). Although functional connectivity analyses were deemed inappropriate for the paradigm used in the current study, future research could explore connectivity between the dlPFC and ventral stream as a potential mechanism underlying the interpersonal model of overeating.

In response to social threat (i.e., angry) cues, girls with LOC-eating may demonstrate aberrant neural circuitry central to food reward and attention allocation (i.e., insula and anterior cingulate) (44–48) that contributes to a maladaptive pattern of eating. Consumption of dessert-type foods, typically high in carbohydrates, is often sought out as “comfort foods” to reduce negative effects and may offer high reward value. Laboratory test meal studies have found that youth demonstrate greater consumption of highly palatable foods during LOC-eating episodes, such as carbohydrates (56). Prospectively, greater consumption of foods high in carbohydrates or fats may serve as a mechanism for excess weight gain or development of full-syndrome binge-eating disorder (13, 58). Furthermore, self-reported negative effect has predicted a greater percentage of intake from carbohydrates in the laboratory (90, 91). The pattern of neural activation and eating observed in the current study supports a potential mechanism aligning with this LOC-eating literature. However, given the marginal significance of interactions and a large number of tests performed, as well as the large and multifunctional nature of the implicated ROIs, it is uncertain whether these interactions would replicate in larger samples. The findings concerning relations between neural responses and energy intake patterns should be considered inconclusive and interpreted with caution.

The current study has several strengths. First, the sample consisted of a racially and ethnically diverse subset of adolescent girls with overweight or obesity, which improves the representativeness and generalizability of results. A well-validated semi-structured interview was used to assess LOC-eating (10, 61, 92). MEG is an ideally suited neuroimaging methodology for the temporal nature of attention bias to social threat and short-occurring activity in neural circuits involved in threat processing (52–54). The inclusion of relevant covariates improves the internal validity of the findings, and body composition covariates are objectively measured by trained staff using calibrated scales and stadiometers for height and weight, and DXA techniques measure fat mass. Finally, the laboratory test meal is a well-validated and controlled paradigm (56, 91) that allowed for objective analysis of energy intake among youth with and without LOC eating in a highly controlled environment.

This study has several notable limitations. First, due to social distancing requirements and stay-at-home orders during the COVID-19 pandemic, recruitment was truncated, resulting in an underpowered sample for most analyses. This recruitment barrier indicates that all results should be interpreted with caution. Furthermore, although consistent with attrition rates in other MEG studies of youth (55, 59), 38% of participants had missing or unusable data. Additionally, the design of the larger pilot study restricted the sample only to female adolescents with overweight and obesity. A comparison group of youth with average BMI, adolescent boys, or younger, pre-pubertal children would aid future studies in terms of generalizability, given the vast differences between boys and girls in nutritional needs across the various stages of the developmental spectrum (93). Given the odds of obesity are higher among youth identifying as Hispanic/Latino and non-Hispanic Black compared with non-Hispanic white youth (1), this underrepresented group may be a particularly vulnerable population in need of further study. Moreover, given the sample is comprised entirely of girls with overweight or obesity, it cannot be determined how specific neural findings might differentially manifest among youth with an average BMI percentile, thus limiting the generalizability. Indeed, neuroimaging findings among adolescent girls with overweight or obesity demonstrate elevated reward region response to palatable food cues and food receipt compared with controls of normal weight, while the risk of eating pathology even further enhanced reward region responsivity to food cues (94). While the current study focused on a small portion of information processing functions linked to LOC-eating, future studies might consider alternate neural mechanisms (e.g., inhibitory control and reward processing). Additional limitations include the use of cross-sectional data; as such, no causal conclusions can be drawn from the findings. The laboratory buffet test meal may not have accurately reflected eating in the natural environment, potentially limiting the ecological validity of the findings. Youths' natural social environments, which can involve salient social threat cues such as weight-based teasing and bullying (95–97), may provide further insights into the links between social threat and LOC-eating behaviors among girls with overweight or obesity. Although the current test meal included foods typically considered palatable by youth (56), it is possible that the composition of the laboratory buffet test meal used in the current study may have impacted the pattern of macronutrient findings. For example, the current test meal included some dessert-type foods that were high in carbohydrate but low in fat (e.g., jelly beans), as opposed to highly palatable dessert-type foods that are high in both carbohydrates and fats (e.g., ice cream) (56). Finally, it cannot be determined to what extent the social threat task influenced experienced social exclusion among youth, as rejection sensitivity was not assessed. This should be considered in future studies along with the other aforementioned limitations.

In conclusion, evoked response patterns in visual and cognitive control neural regions among adolescent girls with overweight or obesity may support the interpersonal model of LOC-eating. Hypoactivation in higher order executive function regions in response to social threat cues may contribute to disinhibited eating patterns that could lead to excess weight gain. Sustained visual attention bias to social threat cues may elucidate an underlying neural mechanism for LOC-eating and could inform early treatment targets for the prevention of excess weight gain.

## Data availability statement

The original contributions presented in the study are included in the article/Supplementary material, further inquiries can be directed to the corresponding authors.

## Ethics statement

The studies involving humans were approved by the Institutional Review Board of the Eunice Kennedy Shriver National Institute of Child Health and Human Development, National Institutes of Health. The studies were conducted in accordance with the local legislation and institutional requirements. Written informed consent for participation in this study was provided by the participants' legal guardians/next of kin.

## Author contributions

MB: Conceptualization, Funding acquisition, Methodology, Project administration, Data curation, Formal analysis, Investigation, Software, Validation, Visualization, Writing—original draft. MT-K: Conceptualization, Funding acquisition, Methodology, Resources, Supervision, Writing—review and editing. LL: Conceptualization, Data curation, Formal analysis, Software, Validation, Visualization, Writing—original draft. TH: Data curation, Software, Writing—review and editing. MP: Writing—review and editing, Investigation. BB: Investigation, Writing—review and editing. AN: Writing—review and editing, Conceptualization, Resources. SB: Writing—review and editing, Investigation. SY: Investigation, Writing—review and editing. ST: Investigation, Writing—review and editing. DP: Writing—review and editing, Conceptualization, Funding acquisition, Resources, Supervision. JY: Conceptualization, Funding acquisition, Resources, Supervision, Writing—review and editing, Methodology, Project administration.
