# Supplementary material for: Neural underpinnings of threat bias in relation to loss-of-control eating behaviors among adolescent girls with high weight
Source: Front Psychiatry. 2023 Oct 27;14:1276300. doi: 10.3389/fpsyt.2023.1276300 (PMC10642175; doi:10.3389/fpsyt.2023.1276300)
Supplement: Supplementary file 1 [file Data_Sheet_1.PDF]

Supplementary Material

Neural underpinnings of threat bias in relation to loss-of-control eating behaviors among adolescent girls with high weight

Meghan E. Byrne [1,2], Marian Tanofsky-Kraff [2,3,4*], Lucrezia Liuzzi[1], Tom Holroyd[5], Megan N. Parker[2,3], Bess F. Bloomer[2], Allison Nugent[5], Sheila M. Brady[2], Shanna B. Yang[6], Sara A. Turner[6], Daniel S. Pine[1], & Jack A. Yanovski[2*]

[1] Section on Development and Affective Neuroscience, Emotion and Development Branch, National Institute of Mental Health (NIMH), Bethesda, MD, USA

[2] Section on Growth and Obesity, *Eunice Kennedy Shriver* National Institute of Child Health and Human Development (NICHD), Division of Intramural Research, National Institutes of Health (NIH), 10 Center Drive, Bethesda, MD, 20892, USA

[3] Department of Medical and Clinical Psychology, USUHS, 4301 Jones Bridge Road, Bethesda, MD, 20814, USA

[4] Military Cardiovascular Outcomes Research (MiCOR) Program, Department of Medicine, Uniformed Services University of the Health Sciences (USUHS), 4301 Jones Bridge Road, Bethesda, MD, 20814, USA

[5] MEG Core Facility, National Institute of Mental Health (NIMH), Bethesda, MD, USA

[6] Nutrition Department, Clinical Center, NIH, 10 Center Drive, Bethesda, MD, 20892, USA

*** Correspondence:**
Jack A. Yanovski, MD, PhD
Section on Growth and Obesity, *Eunice Kennedy Shriver* National Institute of Child Health and Human Development, National Institutes of Health, 10 Center Drive, Room 1-3330, MSC 1103, Bethesda, MD, 20892-1103, Phone: 301-496-0858; FAX 301-480-4271, Email: yanovskj@mail.nih.gov

Marian Tanofsky-Kraff, PhD
Departments of Medical and Clinical Psychology and Medicine, Uniformed Services University of the Health Sciences, 4301 Jones Bridge Road, Bethesda, MD 20814-4712, Phone: 301-295-1482, Fax: 301-400-4296, Email: marian.tanofsky-kraff@usuhs.edu

1 **Supplementary Table 1. Freesurfer (aparc.a2009s+aseg.mgz) regions-of-interest (ROI).**

ROI		Freesurfer ROI label
Amygdala	Left Amygdala	Left-Amygdala
	Right Amygdala	Right-Amygdala
Striatum	Left Striatum	Left-Caudate<br>Left-Putamen<br>Left-Pallidum
	Right Striatum	Right-Caudate<br>Right-Putamen<br>Right-Pallidum
Insula	Left Insula	ctx_lh_G_insular_short
	Right Insula	ctx_rh_G_insular_short
ACC	Left ACC	ctx_lh_G_and_S_cingul-Ant<br>ctx_lh_G_and_S_cingul-Mid-Ant
	Right ACC	ctx_rh_G_and_S_cingul-Ant<br>ctx_rh_G_and_S_cingul-Mid-Ant
Fusiform	Left Fusiform Gyrus	ctx_lh_G_oc-temp_lat-fusifor
	Right Fusiform Gyrus	ctx_rh_G_oc-temp_lat-fusifor
Occipital	Left Occipital	ctx_lh_Pole_occipital<br>ctx_lh_G_occipital_middle
	Right Occipital	ctx_rh_Pole_occipital<br>ctx_rh_G_occipital_middle
mPFC	Left mPFC	ctx_lh_G_front_sup
	Right mPFC	ctx_rh_G_front_sup
dlPFC	Left dlPFC	ctx_lh_S_front_sup<br>ctx_lh_G_front_middle
	Right dlPFC	ctx_rh_S_front_sup<br>ctx_rh_G_front_middle
vlPFC	Left vlPFC	ctx_lh_G_orbital<br>ctx_lh_Lat_Fis-ant-Vertical<br>ctx_lh_G_front_inf-Opercular<br>ctx_lh_G_front_inf-Triangul<br>ctx_lh_G_front_inf-Orbital
	Right vlPFC	ctx_rh_G_orbital<br>ctx_rh_Lat_Fis-ant-Vertical<br>ctx_rh_G_front_inf-Opercular<br>ctx_rh_G_front_inf-Triangul<br>ctx_rh_G_front_inf-Orbital

Note. ACC = anterior cingulate cortex; mPFC = medial prefrontal cortex; dlPFC = dorsolateral prefrontal cortex; vlPFC = ventrolateral prefrontal cortex.

2 **Supplementary Table 2. Generalized Linear Mixed Model of LOC-Eating and Neural Evoked Response on Test Meal Carbohydrate Intake**

**Dependent Variable: % Energy Consumed from Carbohydrates**	**F**	***p***
LeftStriatum_Abu	0.077	0.784
LOC_presence	3.086	0.093
LOC_presence * LeftStriatum_Abu	2.196	0.153
RightStriatum_Abu	0.000	0.984
LOC_presence	0.346	0.563
LOC_presence * RightStriatum_Abu	0.004	0.953
LeftInsula_Abu	0.173	0.681
LOC_presence	5.964	0.023
LOC_presence * LeftInsula_Abu	5.563	0.028
RightInsula_Abu	0.011	0.919
LOC_presence	0.366	0.551
LOC_presence * RightInsula_Abu	0.006	0.940
LeftAmygdala_Abu	1.533	0.229
LOC_presence	4.169	0.053
LOC_presence * LeftAmygdala_Abu	3.689	0.068
RightAmygdala_Abu	0.446	0.511
LOC_presence	1.348	0.258
LOC_presence * RightAmygdala_Abu	0.605	0.445
LeftACC_Abu	2.926	0.101
LOC_presence	0.228	0.638
LOC_presence * LeftACC_Abu	1.498	0.234
RightACC_Abu	5.133	0.034
LOC_presence	4.152	0.054
LOC_presence * RightACC_Abu	2.908	0.102
LeftvlPFC_Abu	2.493	0.129
LOC_presence	3.161	0.089
LOC_presence * LeftvlPFC_Abu	3.805	0.064
RightvlPFC_Abu	0.029	0.866
LOC_presence	0.029	0.866
LOC_presence * RightvlPFC_Abu	0.224	0.640
LeftdlPFC_Abu	0.015	0.905
LOC_presence	0.855	0.365
LOC_presence * LeftdlPFC_Abu	0.050	0.825
RightdlPFC_Abu	0.006	0.941
LOC_presence	0.000	0.999
LOC_presence * RightdlPFC_Abu	1.804	0.193
LeftmPFC_Abu	2.222	0.150
LOC_presence	0.110	0.743
LOC_presence * LeftmPFC_Abu	2.718	0.113
RightmPFC_Abu	0.116	0.737
LOC_presence	0.000	0.986

LOC_presence * RightmPFC_Abu	1.001	0.328
LeftOccipital_Abu	0.012	0.913
LOC_presence	0.010	0.920
LOC_presence * LeftOccipital_Abu	0.361	0.554
RightOccipital_Abu	5.518	0.028
LOC_presence	0.208	0.653
LOC_presence * RightOccipital_Abu	0.404	0.531
LeftFusiform_Abu	0.002	0.965
LOC_presence	0.226	0.639
LOC_presence * LeftFusiform_Abu	0.138	0.713
RightFusiform_Abu	1.184	0.288
LOC_presence	0.071	0.792
LOC_presence * RightFusiform_Abu	1.657	0.211
LeftStriatum_Atd	0.006	0.939
LOC_presence	0.392	0.537
LOC_presence * LeftStriatum_Atd	0.164	0.689
RightStriatum_Atd	1.536	0.228
LOC_presence	1.189	0.287
LOC_presence * RightStriatum_Atd	0.613	0.442
LeftInsula_Atd	1.514	0.232
LOC_presence	1.353	0.257
LOC_presence * LeftInsula_Atd	0.362	0.553
RightInsula_Atd	0.231	0.636
LOC_presence	1.127	0.300
LOC_presence * RightInsula_Atd	0.551	0.466
LeftAmygdala_Atd	0.304	0.587
LOC_presence	0.972	0.335
LOC_presence * LeftAmygdala_Atd	0.499	0.487
RightAmygdala_Atd	0.003	0.960
LOC_presence	0.947	0.341
LOC_presence * RightAmygdala_Atd	0.174	0.681
LeftACC_Atd	0.908	0.351
LOC_presence	1.703	0.205
LOC_presence * LeftACC_Atd	8.228	**0.009***
RightACC_Atd	0.043	0.838
LOC_presence	0.003	0.955
LOC_presence * RightACC_Atd	1.683	0.208
LeftvlPFC_Atd	0.165	0.688
LOC_presence	0.381	0.543
LOC_presence * LeftvlPFC_Atd	0.007	0.933
RightvlPFC_Atd	1.047	0.317
LOC_presence	0.004	0.949
LOC_presence * RightvlPFC_Atd	1.320	0.263
LeftdlPFC_Atd	2.672	0.116
LOC_presence	2.170	0.155

LOC_presence * LeftdlPFC_Atd	1.351	0.258
RightdlPFC_Atd	1.411	0.248
LOC_presence	1.733	0.202
LOC_presence * RightdlPFC_Atd	0.818	0.375
LeftmPFC_Atd	2.228	0.150
LOC_presence	0.006	0.940
LOC_presence * LeftmPFC_Atd	1.696	0.206
RightmPFC_Atd	0.444	0.512
LOC_presence	0.526	0.476
LOC_presence * RightmPFC_Atd	0.013	0.912
LeftOccipital_Atd	0.559	0.463
LOC_presence	0.387	0.540
LOC_presence * LeftOccipital_Atd	6.142	0.021
RightOccipital_Atd	0.512	0.482
LOC_presence	0.071	0.793
LOC_presence * RightOccipital_Atd	1.600	0.219
LeftFusiform_Atd	1.358	0.256
LOC_presence	1.244	0.277
LOC_presence * LeftFusiform_Atd	0.143	0.709
RightFusiform_Atd	0.455	0.507
LOC_presence	0.180	0.675
LOC_presence * RightFusiform_Atd	1.787	0.195
LeftStriatum_Hbu	2.354	0.139
LOC_presence	0.442	0.513
LOC_presence * LeftStriatum_Hbu	0.006	0.937
RightStriatum_Hbu	0.969	0.336
LOC_presence	0.101	0.753
LOC_presence * RightStriatum_Hbu	0.952	0.340
LeftInsula_Hbu	2.709	0.114
LOC_presence	0.219	0.645
LOC_presence * LeftInsula_Hbu	0.447	0.511
RightInsula_Hbu	2.188	0.153
LOC_presence	1.514	0.232
LOC_presence * RightInsula_Hbu	0.065	0.800
LeftAmygdala_Hbu	1.238	0.278
LOC_presence	1.359	0.256
LOC_presence * LeftAmygdala_Hbu	0.034	0.856
RightAmygdala_Hbu	0.032	0.859
LOC_presence	1.067	0.313
LOC_presence * RightAmygdala_Hbu	0.157	0.696
LeftACC_Hbu	0.257	0.618
LOC_presence	2.606	0.121
LOC_presence * LeftACC_Hbu	1.731	0.202
RightACC_Hbu	0.005	0.945
LOC_presence	1.731	0.202

LOC_presence * RightACC_Hbu	0.623	0.438
LeftvlPFC_Hbu	0.309	0.584
LOC_presence	0.610	0.443
LOC_presence * LeftvlPFC_Hbu	0.001	0.977
RightvlPFC_Hbu	0.240	0.629
LOC_presence	1.327	0.262
LOC_presence * RightvlPFC_Hbu	0.199	0.660
LeftdlPFC_Hbu	0.004	0.952
LOC_presence	0.649	0.429
LOC_presence * LeftdlPFC_Hbu	0.028	0.868
RightdlPFC_Hbu	2.182	0.154
LOC_presence	0.536	0.472
LOC_presence * RightdlPFC_Hbu	0.752	0.395
LeftmPFC_Hbu	1.040	0.319
LOC_presence	1.217	0.282
LOC_presence * LeftmPFC_Hbu	1.081	0.310
RightmPFC_Hbu	1.482	0.236
LOC_presence	1.398	0.250
LOC_presence * RightmPFC_Hbu	1.541	0.228
LeftOccipital_Hbu	0.005	0.943
LOC_presence	1.242	0.277
LOC_presence * LeftOccipital_Hbu	0.267	0.611
RightOccipital_Hbu	1.146	0.296
LOC_presence	6.757	0.016
LOC_presence * RightOccipital_Hbu	7.717	0.011
LeftFusiform_Hbu	0.564	0.461
LOC_presence	3.191	0.088
LOC_presence * LeftFusiform_Hbu	3.716	0.067
RightFusiform_Hbu	0.001	0.972
LOC_presence	1.069	0.312
LOC_presence * RightFusiform_Hbu	0.159	0.694
LeftStriatum_Htd	0.357	0.556
LOC_presence	1.248	0.276
LOC_presence * LeftStriatum_Htd	0.030	0.864
RightStriatum_Htd	0.797	0.382
LOC_presence	0.581	0.454
LOC_presence * RightStriatum_Htd	0.120	0.733
LeftInsula_Htd	0.894	0.355
LOC_presence	1.121	0.301
LOC_presence * LeftInsula_Htd	0.057	0.814
RightInsula_Htd	1.094	0.307
LOC_presence	0.734	0.401
LOC_presence * RightInsula_Htd	0.042	0.840
LeftAmygdala_Htd	1.779	0.196
LOC_presence	0.805	0.379

LOC_presence * LeftAmygdala_Htd	0.040	0.844
RightAmygdala_Htd	0.016	0.901
LOC_presence	2.139	0.158
LOC_presence * RightAmygdala_Htd	1.887	0.183
LeftACC_Htd	0.026	0.873
LOC_presence	1.359	0.256
LOC_presence * LeftACC_Htd	0.189	0.668
RightACC_Htd	0.767	0.391
LOC_presence	1.936	0.178
LOC_presence * RightACC_Htd	1.419	0.246
LeftvlPFC_Htd	0.864	0.363
LOC_presence	0.376	0.546
LOC_presence * LeftvlPFC_Htd	0.143	0.709
RightvlPFC_Htd	0.270	0.608
LOC_presence	1.232	0.279
LOC_presence * RightvlPFC_Htd	0.082	0.777
LeftdlPFC_Htd	6.215	0.021
LOC_presence	0.004	0.951
LOC_presence * LeftdlPFC_Htd	4.876	0.038
RightdlPFC_Htd	0.107	0.746
LOC_presence	0.572	0.458
LOC_presence * RightdlPFC_Htd	0.018	0.894
LeftmPFC_Htd	0.127	0.725
LOC_presence	1.225	0.280
LOC_presence * LeftmPFC_Htd	0.469	0.501
RightmPFC_Htd	0.257	0.617
LOC_presence	0.289	0.596
LOC_presence * RightmPFC_Htd	0.497	0.488
LeftOccipital_Htd	0.002	0.965
LOC_presence	0.913	0.350
LOC_presence * LeftOccipital_Htd	0.017	0.897
RightOccipital_Htd	0.625	0.438
LOC_presence	1.222	0.281
LOC_presence * RightOccipital_Htd	0.399	0.534
LeftFusiform_Htd	0.284	0.599
LOC_presence	1.295	0.267
LOC_presence * LeftFusiform_Htd	0.002	0.966
RightFusiform_Htd	3.974	0.059
LOC_presence	3.794	0.064
LOC_presence * RightFusiform_Htd	3.778	0.065

Note. Abu = Angry face cue bottom-up time window (0-250 ms) average evoked response; Atd = Angry face cue top-down time window (250-600 ms) average evoked response; Hbu = Happy face cue bottom-up time window (0-250 ms) average evoked response; Htd = Happy face cue top-down time window (250-600 ms) average evoked response.

3 **Supplementary Table 3. Generalized Linear Mixed Model of LOC-Eating and Neural Evoked Response on Test Meal Fat Intake**

Dependent Variable: % Energy Consumed from Fats	F	*p*
LeftStriatum_Abu	0.537	0.471
LOC_presence	4.227	0.052
LOC_presence * LeftStriatum_Abu	3.543	0.073
RightStriatum_Abu	0.335	0.569
LOC_presence	0.188	0.669
LOC_presence * RightStriatum_Abu	0.004	0.952
LeftInsula_Abu	0.004	0.951
LOC_presence	7.715	0.011
LOC_presence * LeftInsula_Abu	8.186	**0.009***
RightInsula_Abu	0.289	0.597
LOC_presence	0.371	0.549
LOC_presence * RightInsula_Abu	0.001	0.980
LeftAmygdala_Abu	0.307	0.585
LOC_presence	3.908	0.061
LOC_presence * LeftAmygdala_Abu	3.148	0.090
RightAmygdala_Abu	0.802	0.380
LOC_presence	2.140	0.158
LOC_presence * RightAmygdala_Abu	1.460	0.240
LeftACC_Abu	3.653	0.069
LOC_presence	0.009	0.927
LOC_presence * LeftACC_Abu	0.410	0.529
RightACC_Abu	4.417	0.047
LOC_presence	4.826	0.039
LOC_presence * RightACC_Abu	3.698	0.068
LeftvlPFC_Abu	1.278	0.271
LOC_presence	3.569	0.072
LOC_presence * LeftvlPFC_Abu	4.006	0.058
RightvlPFC_Abu	0.085	0.774
LOC_presence	0.611	0.443
LOC_presence * RightvlPFC_Abu	0.085	0.773
LeftdlPFC_Abu	0.357	0.556
LOC_presence	0.936	0.344
LOC_presence * LeftdlPFC_Abu	0.055	0.816
RightdlPFC_Abu	0.094	0.762
LOC_presence	0.075	0.786
LOC_presence * RightdlPFC_Abu	0.740	0.399
LeftmPFC_Abu	2.953	0.100
LOC_presence	0.000	0.994
LOC_presence * LeftmPFC_Abu	1.101	0.305
RightmPFC_Abu	0.012	0.913
LOC_presence	0.049	0.828

LOC_presence * RightmPFC_Abu	0.448	0.510
LeftOccipital_Abu	0.151	0.701
LOC_presence	0.080	0.780
LOC_presence * LeftOccipital_Abu	0.073	0.790
RightOccipital_Abu	6.942	0.015
LOC_presence	0.608	0.444
LOC_presence * RightOccipital_Abu	1.385	0.252
LeftFusiform_Abu	0.022	0.884
LOC_presence	0.237	0.631
LOC_presence * LeftFusiform_Abu	0.077	0.783
RightFusiform_Abu	3.505	0.075
LOC_presence	0.001	0.971
LOC_presence * RightFusiform_Abu	0.694	0.414
LeftStriatum_Atd	0.031	0.861
LOC_presence	0.171	0.683
LOC_presence * LeftStriatum_Atd	0.685	0.417
RightStriatum_Atd	0.720	0.405
LOC_presence	1.033	0.320
LOC_presence * RightStriatum_Atd	0.338	0.567
LeftInsula_Atd	2.137	0.158
LOC_presence	1.193	0.287
LOC_presence * LeftInsula_Atd	0.195	0.663
RightInsula_Atd	0.035	0.853
LOC_presence	1.208	0.284
LOC_presence * RightInsula_Atd	0.827	0.373
LeftAmygdala_Atd	0.443	0.513
LOC_presence	0.958	0.338
LOC_presence * LeftAmygdala_Atd	1.031	0.321
RightAmygdala_Atd	0.018	0.894
LOC_presence	0.947	0.341
LOC_presence * RightAmygdala_Atd	0.161	0.692
LeftACC_Atd	1.704	0.205
LOC_presence	1.690	0.207
LOC_presence * LeftACC_Atd	8.046	**0.010***
RightACC_Atd	0.004	0.948
LOC_presence	0.001	0.980
LOC_presence * RightACC_Atd	1.957	0.176
LeftvlPFC_Atd	0.010	0.920
LOC_presence	0.269	0.609
LOC_presence * LeftvlPFC_Atd	0.212	0.650
RightvlPFC_Atd	1.192	0.287
LOC_presence	0.037	0.849
LOC_presence * RightvlPFC_Atd	0.584	0.453
LeftdlPFC_Atd	3.720	0.067
LOC_presence	2.367	0.138

LOC_presence * LeftdlPFC_Atd	1.627	0.215
RightdlPFC_Atd	1.404	0.249
LOC_presence	1.812	0.192
LOC_presence * RightdlPFC_Atd	0.960	0.338
LeftmPFC_Atd	1.870	0.185
LOC_presence	0.001	0.971
LOC_presence * LeftmPFC_Atd	1.195	0.286
RightmPFC_Atd	0.388	0.540
LOC_presence	0.489	0.492
LOC_presence * RightmPFC_Atd	0.018	0.895
LeftOccipital_Atd	0.014	0.908
LOC_presence	0.263	0.613
LOC_presence * LeftOccipital_Atd	4.375	0.048
RightOccipital_Atd	0.098	0.757
LOC_presence	0.094	0.762
LOC_presence * RightOccipital_Atd	0.712	0.408
LeftFusiform_Atd	1.923	0.179
LOC_presence	0.913	0.350
LOC_presence * LeftFusiform_Atd	0.002	0.968
RightFusiform_Atd	0.014	0.906
LOC_presence	0.103	0.751
LOC_presence * RightFusiform_Atd	1.354	0.257
LeftStriatum_Hbu	1.567	0.224
LOC_presence	0.065	0.801
LOC_presence * LeftStriatum_Hbu	0.573	0.457
RightStriatum_Hbu	1.335	0.260
LOC_presence	0.019	0.893
LOC_presence * RightStriatum_Hbu	1.663	0.211
LeftInsula_Hbu	2.511	0.127
LOC_presence	0.050	0.825
LOC_presence * LeftInsula_Hbu	1.284	0.269
RightInsula_Hbu	3.226	0.086
LOC_presence	1.355	0.257
LOC_presence * RightInsula_Hbu	0.003	0.955
LeftAmygdala_Hbu	0.812	0.377
LOC_presence	0.595	0.449
LOC_presence * LeftAmygdala_Hbu	0.124	0.729
RightAmygdala_Hbu	0.000	0.986
LOC_presence	0.808	0.379
LOC_presence * RightAmygdala_Hbu	0.013	0.910
LeftACC_Hbu	0.133	0.719
LOC_presence	1.867	0.186
LOC_presence * LeftACC_Hbu	0.893	0.355
RightACC_Hbu	0.009	0.926
LOC_presence	1.381	0.252

LOC_presence * RightACC_Hbu	0.332	0.570
LeftvlPFC_Hbu	0.706	0.410
LOC_presence	0.268	0.610
LOC_presence * LeftvlPFC_Hbu	0.186	0.671
RightvlPFC_Hbu	0.277	0.604
LOC_presence	1.168	0.292
LOC_presence * RightvlPFC_Hbu	0.102	0.752
LeftdlPFC_Hbu	0.103	0.751
LOC_presence	0.070	0.793
LOC_presence * LeftdlPFC_Hbu	0.273	0.607
RightdlPFC_Hbu	1.422	0.246
LOC_presence	0.372	0.548
LOC_presence * RightdlPFC_Hbu	0.291	0.595
LeftmPFC_Hbu	1.299	0.267
LOC_presence	0.851	0.366
LOC_presence * LeftmPFC_Hbu	0.640	0.432
RightmPFC_Hbu	1.605	0.218
LOC_presence	1.130	0.299
LOC_presence * RightmPFC_Hbu	1.203	0.285
LeftOccipital_Hbu	0.108	0.746
LOC_presence	0.687	0.416
LOC_presence * LeftOccipital_Hbu	0.001	0.970
RightOccipital_Hbu	0.648	0.429
LOC_presence	5.297	0.031
LOC_presence * RightOccipital_Hbu	5.487	0.029
LeftFusiform_Hbu	0.048	0.829
LOC_presence	1.703	0.205
LOC_presence * LeftFusiform_Hbu	1.023	0.323
RightFusiform_Hbu	0.000	0.987
LOC_presence	0.628	0.436
LOC_presence * RightFusiform_Hbu	0.079	0.781
LeftStriatum_Htd	0.340	0.566
LOC_presence	1.026	0.322
LOC_presence * LeftStriatum_Htd	0.001	0.980
RightStriatum_Htd	0.854	0.365
LOC_presence	0.528	0.475
LOC_presence * RightStriatum_Htd	0.151	0.702
LeftInsula_Htd	0.973	0.335
LOC_presence	0.881	0.358
LOC_presence * LeftInsula_Htd	0.205	0.655
RightInsula_Htd	1.234	0.279
LOC_presence	0.758	0.393
LOC_presence * RightInsula_Htd	0.027	0.872
LeftAmygdala_Htd	1.191	0.287
LOC_presence	0.551	0.466

LOC_presence * LeftAmygdala_Htd	0.151	0.702
RightAmygdala_Htd	0.070	0.794
LOC_presence	2.267	0.146
LOC_presence * RightAmygdala_Htd	2.297	0.144
LeftACC_Htd	0.088	0.770
LOC_presence	1.191	0.287
LOC_presence * LeftACC_Htd	0.216	0.647
RightACC_Htd	0.895	0.354
LOC_presence	1.873	0.185
LOC_presence * RightACC_Htd	1.434	0.244
LeftvlPFC_Htd	1.305	0.266
LOC_presence	0.128	0.724
LOC_presence * LeftvlPFC_Htd	0.542	0.469
RightvlPFC_Htd	0.197	0.661
LOC_presence	1.403	0.249
LOC_presence * RightvlPFC_Htd	0.197	0.661
LeftdlPFC_Htd	8.814	**0.007***
LOC_presence	0.013	0.912
LOC_presence * LeftdlPFC_Htd	7.185	0.014
RightdlPFC_Htd	0.228	0.638
LOC_presence	0.459	0.505
LOC_presence * RightdlPFC_Htd	0.072	0.791
LeftmPFC_Htd	0.115	0.738
LOC_presence	0.909	0.351
LOC_presence * LeftmPFC_Htd	0.175	0.680
RightmPFC_Htd	0.308	0.584
LOC_presence	0.239	0.630
LOC_presence * RightmPFC_Htd	0.612	0.442
LeftOccipital_Htd	0.000	0.992
LOC_presence	0.815	0.377
LOC_presence * LeftOccipital_Htd	0.045	0.834
RightOccipital_Htd	0.626	0.437
LOC_presence	1.112	0.303
LOC_presence * RightOccipital_Htd	0.280	0.602
LeftFusiform_Htd	0.728	0.403
LOC_presence	1.142	0.297
LOC_presence * LeftFusiform_Htd	0.119	0.733
RightFusiform_Htd	4.397	0.048
LOC_presence	3.559	0.073
LOC_presence * RightFusiform_Htd	3.393	0.079

Note. Abu = Angry face cue bottom-up time window (0-250 ms) average evoked response; Atd = Angry face cue top-down time window (250-600 ms) average evoked response; Hbu = Happy face cue bottom-up time window (0-250 ms) average evoked response; Htd = Happy face cue top-down time window (250-600 ms) average evoked response.
